# Prognostic factors, oncological treatment and outcomes of uterine sarcoma: 10 years’ clinical experience from a tertiary care center in Pakistan

**DOI:** 10.1186/s12885-023-11000-3

**Published:** 2023-06-05

**Authors:** Saqib Raza Khan, Salman Muhammad Soomar, Tamana Asghari, Arsalan Ahmed, Munira Shabbir Moosajee

**Affiliations:** 1 Department of Oncology, Aga Khan University Hospital, Karachi, Pakistan; 2 Aga Khan University, 74800 Karachi, Pakistan; 3 Department of Histopathology, Department of Pathology and Laboratory Medicine, Aga Khan University Hospital, Karachi, Pakistan

**Keywords:** Uterine sarcoma, Prognosis, Malignancy, Chemotherapy, Survival

## Abstract

**Background:**

Uterine sarcoma is an uncommon aggressive malignancy. Optimal management and prognostic factors have yet to be well recognized due to their rarity and various histological subtypes. This study aims to investigate these patients' prognostic factors, treatment modalities, and oncological outcomes.

**Methods:**

A single-center retrospective cohort study was conducted on all patients diagnosed with uterine sarcoma and treated from January 2010 to December 2019 in a tertiary-care hospital in Pakistan. The data were analyzed using STATA software and stratified on the histological subtype. Survival rates were estimated using the Kaplan–Meier method. Crude and adjusted hazard ratios with 95% CI were estimated using univariate and multivariate analysis.

**Results:**

Of the 40 patients, 16(40%) had uterine leiomyosarcoma (u-LMS), 10(25%) had high-grade endometrial stromal sarcoma (HGESS), 8(20%) had low-grade endometrial stromal sarcoma (LGESS) and 6(15%) had other histological subtypes. The median age of all patients was 49 (40–55.5). Thirty-seven (92.5%) patients underwent primary surgical resection, and 24 (60%) patients received adjuvant systemic chemotherapy. The survival plots showed the overall population's DFS of 64 months and the OS of 88 months (*p*-value = 0.001). The median DFS in all patients was 12 months, and the median OS was 14 months (*p*-value = 0.001). A small but significant DFS benefit was found in patients who received adjuvant systemic chemotherapy, 13.5 versus 11 months (*p*-value = 0.001). Multivariate Cox-regression analysis revealed that large tumor size and advanced FIGO stage were substantial factors associated with decreased survival.

**Conclusion:**

Uterine sarcomas are rare malignancies with poor prognosis. Multiple factors, including tumor size, mitotic count, stage of the disease, and myometrial invasion, impact survival outcomes. Adjuvant treatment may decrease the recurrence rate and improve DFS but do not affect OS.

## Introduction

Uterine sarcoma (US) is an uncommon aggressive malignancy of the uterine corpus that accounts for 3–9% of all uterine cancers [1]. Based on histological features, it is further classified into mesenchymal tumors or mixed epithelial and mesenchymal tumors. Mesenchymal tumors are classified as uterine leiomyosarcoma (u-LMS, 63%), high-grade endometrial stromal sarcoma (HGESS), and low-grade endometrial stromal sarcoma, which collectively constitute 21%, undifferentiated uterine sarcoma (UUS, 5%), adenosarcomas (AS, 6%) and some other rare types (5%) [2]. In addition, the National Comprehensive Cancer Network (NCCN) guidelines no longer consider carcinosarcoma as uterine sarcoma because of its epithelial origin [3].

Distinct features of u-LMS are that it usually presents as a bulky tumor, common in women > 40 years of age, and high grade with typically a mitotic rate of > 15/10HPF. The prognosis is despicable and has a high recurrence rate range between 53 to 71% [4]. According to SEER paper reports, a 5-year disease-specific survival of u-LMS for the early stage is 60–70%, while for the advanced stage, it is 29% [5]. LGESS is estrogen and progesterone receptor positive and usually occurs in women > 40 years. The overall prognosis is favorable. Five-year survival for the early stage is 89%, compared to 50% for the advanced stage. Recurrence occurs in one-third of the patients [6]. HGESS is usually estrogen and progesterone receptor negative and has a high recurrence rate, which occurs earlier after primary diagnosis. The SEER database reported the overall survival for all low and high-grade ESS stages as 72.7% [7]. AS are characterized by two components, i.e., benign epithelial elements and malignant mesenchymal ones. Most patients are present in an early stage with a 5–year overall survival of 60–80% [8]. UUS is usually poorly differentiated and presents as stage III-IV disease in 60% of the patients. The prognosis is poor, with a median survival of less than a year when metastasized [9].

Optimal management and prognostic factors have yet to be well recognized due to their rarity and various histological subtypes [10]. Several studies reported that mitotic figures and tumor size are the most important prognostic factors [11, 12]. Total abdominal hysterectomy (TAH) with bilateral salpingo-oophorectomy is the standard primary treatment for uterine sarcoma. The pelvic lymph node dissection role is still under consideration [13]. Numerous studies reported a pelvic lymph node involvement rate of up to 47%, while no significant survival benefit was reported after pelvic lymph node dissection [14]. The role of adjuvant treatment in patients with uterine sarcoma is very limited. A systematic review reported no survival benefit of adjuvant chemotherapy in u-LMS [15]. SEER database also reported no survival benefit of adjuvant radiotherapy in uterine sarcoma [7].

The worldwide incidence is 0.36/100.000 woman-years [1, 16]. Unfortunately, no local study has been done to see the prognosis and oncological outcome of uterine sarcoma; in Pakistan. Therefore, this retrospective study aims to investigate the prognostic factors and identify optimal treatment modalities and oncological outcomes for patients with uterine sarcoma. Institutional guidelines can be proposed based on this analysis.

## Methods

**Fig. 1:**
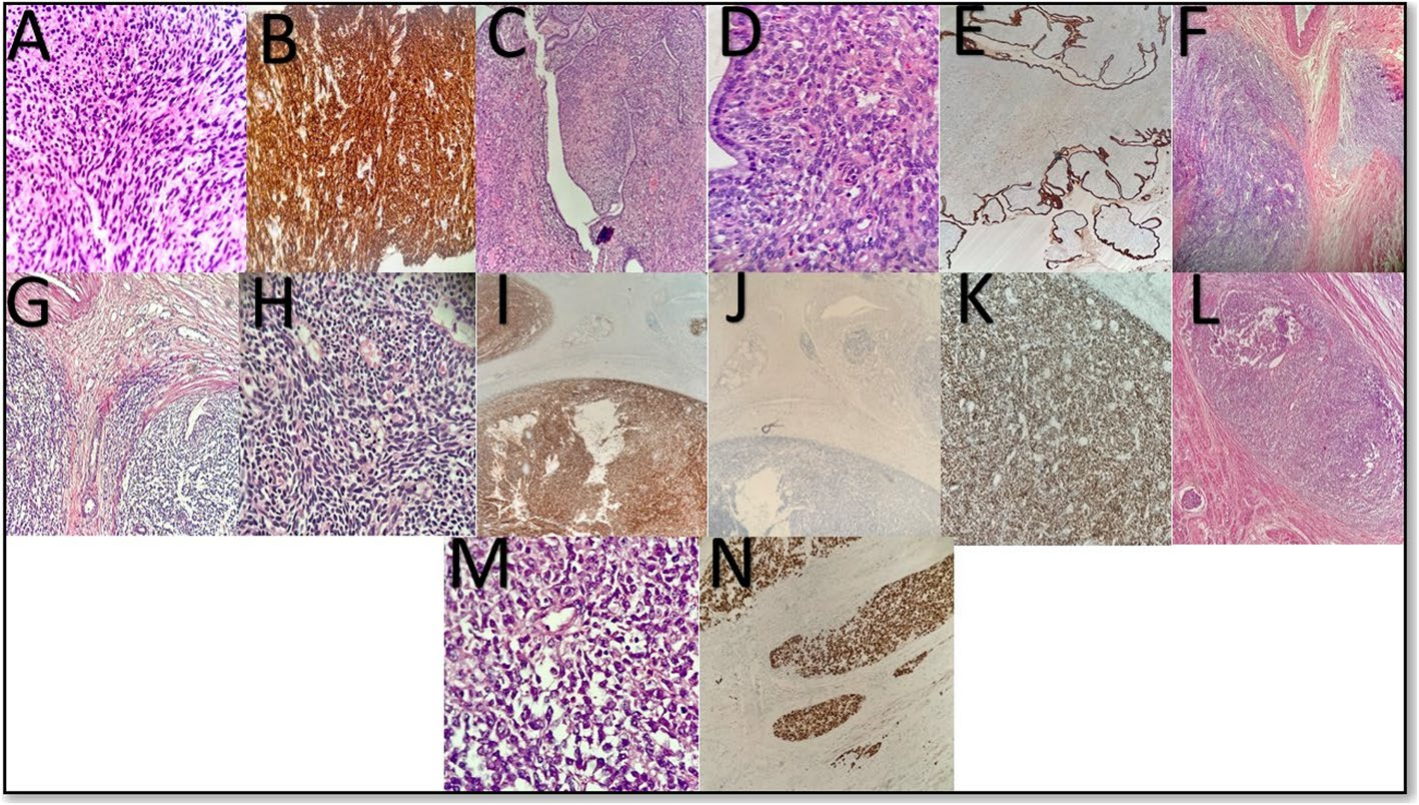
Uterine Leiomyosarcoma (uLMS) (**A**-**B**): H&E shows spindle-shaped cells in fascicles with atypical hyperchromatic nuclei at 40x (**A**). The tumor cells stain diffuse and strong for immunohistochemical (IHC) stain h-caldesmon (**B**). Adenosarcoma(AS) (**C**-**E**): 10x: Characteristic leaf-like architecture of benign glands with surrounding condensed peri glandular stroma (**C**), 40x: Benign glandular epithelium and malignant stroma with cytologic atypia and mitosis (inset) (**D**), The glandular epithelium stains positive for IHC stain Cytokeratin AE1/AE3 (**E**). Low-grade endometrial stromal sarcoma (LGESS) (**F**-**K**): 10x: the lesion shows a permeative tongue like the pattern of myometrial invasion (**F**). At 20 × view the tumor cells appear monotonous ovoid to spindle shape (**G**), High power at 40 × shows minimal cytological atypia with cells whorling around delicate blood vessels (**H**), Diffuse positivity for IHC stain CD10 (**I**), The cells are completely negative for IHC stain Cyclin D1 (**J**), IHC stain-ER diffuse and strong nuclear positive (**K**). High-grade endometrial stromal sarcoma (HGESS) (**L**-**N**): 20x: Nested growth of round cells with scant cytoplasm and lymphovascular invasion (arrow) (**L**), 40x: Cells with scant cytoplasm and nuclear atypia (**M**), IHC stain Cyclin D1 diffuse and strong positive in tumor cells (**N**)

We used a retrospective cohort study design to enroll patients diagnosed with uterine sarcoma and treated between January 2010 to December 2019. The study was conducted at the medical oncology department in a large tertiary care hospital in Karachi, Pakistan. Out of 51 biopsy-proven uterine sarcoma patients, 40 were included in the study. Non-probability purposive sampling was applied for the cohort selection. All histological samples from the records underwent a detailed review by a specialized pathologist and diagnosis remains the same. Patients aged 18 and above and with biopsy-proven uterine sarcoma per the WHO classification of uterine sarcoma 2020 [3, 17] were included. Patients diagnosed with uterine carcinosarcoma, also known as malignant mixed Mullerian tumors (MMMT), were excluded because these tumors are now classified as uterine carcinoma. Patients with metastatic sarcoma from other gynecological sides, patients with distant disease at presentation (FIGO stage IVB), and patients with incomplete information regarding pathological diagnosis, clinical findings, and follow-up studies for analysis, as they were lost to follow up after initial diagnosis, were also excluded. All patients were classified as per the NCCN classification of uterine mesenchymal stromal tumors [3] into four histological subgroups, including uterine leiomyosarcoma (u-LMS), high-grade endometrial stromal sarcoma (HGESS), Low-grade endometrial stromal sarcoma (LGESS), and others (undifferentiated uterine sarcoma and adenosarcoma) (Fig. 1). In addition, all patients were staged according to the current International Federation of Gynecology and Obstetrics (FIGO) criteria [18, 19].

All records were screened for complete medical history and clinical data. The main characteristics of the patients that were assessed and recorded include age, marital status, family history of malignancy, medical illness, menstrual history, parity, and preoperative endometrial biopsy. The main characteristics of the tumor that are recorded and assessed include the histologic grade, tumor size, myometrial invasion, mitotic index, lymphovascular invasion (LVI), lymph node involvement, history of pelvic wash, and stage of the disease. Treatment and outcome variables that were assessed and recorded include type of primary surgery performed, residual disease status, adjuvant chemotherapy received, chemotherapeutic regimen, number of chemotherapy cycles, history of radiation therapy received, hormonal treatment, disease recurrence, and follow-up in the context of the number of patients alive with and without disease and number of patients died.

All patients underwent baseline imaging studies with Computed tomography (CT) scan of the abdomen and pelvis with or without CT chest or Magnetic resonance imaging (MRI) of the pelvis either within four weeks before surgery or four to six weeks after surgery as per the decision of the primary surgeon. In addition, scans were performed in patients who received adjuvant systemic chemotherapy eight to twelve weeks after administering the last dose. Among these, a few patients also underwent disease evaluation by scans during the treatment after receiving three or four cycles of systemic chemotherapy.

Follow-up scans were also performed and documented, and the record was assessed retrospectively. These follow-up scans were performed every three to six months during the initial years, with subsequent scans every six to twelve months during later years of life. Disease-free survival (DFS) is the months from diagnosis to disease recurrence or last follow-up. Overall survival (OS) was defined as the time in months between diagnosis to the date of death or last follow-up.

## Statistical analysis

The data were analyzed using STATA software. Median and IQR were calculated for skewed quantitative variables such as age, and normality was checked through the Shapiro–Wilk test. Frequencies (n) and percentages (%) were calculated for categorical variables such as demographic and clinical characteristics. Data were stratified on histological type, the post-stratification Chi-Square test was used to compare the categorical variables, and the One-way ANOVA test was used to compare the continuous variables. Survival rates were estimated using the Kaplan–Meier method, and survival curves were compared using a log-rank test. Crude and adjusted hazard ratios (HR) with 95% Confidence Intervals (CIs) were estimated using univariate and multivariate analysis. Multivariable cox regression analysis scrutinized the effect on DFS and OS after adjustment for known prognostic baseline variables. *P*-value ≤ 0.05 was considered statistically significant.

## Results

Out of 51 eligible patients with uterine sarcoma, 40 were included in our study analysis. Sixteen patients had uterine leiomyosarcoma (u-LMS), ten patients had high-grade endometrial stromal sarcoma (HGESS), eight patients had low-grade endometrial stromal sarcoma (LGESS), six patients had other histological subtypes (2 with undifferentiated uterine sarcoma [UUS], 4 with adenosarcoma [AS]). The patient's and tumor characteristics are summarized in Table 1.

**Table 1:** Baseline and Tumor characteristics of uterine sarcoma patients stratified on the histological type (*n* = 40)

Characteristics	All Patients (*n* = 40)	u-LMS (*n* = 16)	HGESS (*n* = 10)	LGESS (*n* = 8)	Other histologic subtypes (*n* = 6)	*p*-value
Age in years (median, IQR)	49 (40–55.5)	43 (39.5- 52)	53.5 (48–58)	42.5 (37.5–55.5)	56.5 (45–70)	< 0.001*
Marital status, n(%)						
Single	5 (12.50)	4 (80.0)	0 (0.0)	0 (0.0)	1 (20.0)	0.175
Married	35 (87.50)	12 (34.29)	10 (28.57)	8 (22.86)	5 (14.29)	
Family History of cancer, n(%)						
Yes	2 (5.0)	1 (50.0)	0 (100.0)	0 (100.0)	1 (50.0)	0.437
No	38 (95.0)	15 (39.47)	10 (26.32)	8 (21.05)	5 (13.16)	
Medical illness, n(%)						
DM	10 (25.0)	1 (10.0)	4 (40.0)	2 (20.0)	3 (30.0)	0.176
HTN	8 (20.0)	5 (62.50)	2 (25.00)	0 (0.0)	1 (1.0)	
None	22 (55.0)	10 (45.45)	4 (18.18)	6 (27.27)	2 (9.09)	
Menstrual status, n(%)						
Premenopausal	17 (42.50)	8 (47.06)	1 (5.88)	6 (35.29)	2 (11.76)	0.039
Postmenopausal	23 (57.50)	8 (34.78)	9 (39.13)	2 (8.70)	4 (17.39)	
Parity, n(%)						
Parity = 0	3 (8.57)	0 (0.0)	0 (0.0)	3 (42.86)	0 (0.0)	0.004
Parity ≥ 1	37 (91.43)	14 (43.75)	9 (28.13)	4 (12.50)	4 (15.63)	
Pre-op endometrial biopsy, n(%)						
Positive	13 (32.50)	4 (30.77)	4 (30.77)	2 (15.38)	3 (23.08)	0.534
Negative	1 (2.50)	0 (0.0)	1 (100.0)	0 (0.0)	0 (0.0)	
Not performed / Not documented	26 (65.0)	12 (46.15)	5 (19.23)	6 (23.08)	3 (11.54)	
FIGO Stage, n(%)						
I	16 (40.0)	9 (56.25)	4 (25.0)	2 (12.50)	1 (6.25)	
II	8 (20.0)	1 (12.50)	4 (50.0)	3 (37.50)	0 (0.0)	0.034
III	5 (12.50)	1 (20.0)	0 (0.0)	1 (20.0)	3 (60.0)	
IV	3 (7.50)	3 (100.0)	0 (0.0)	0 (0.0)	0 (0.0)	
Missing/Incomplete data	8 (20.0)	2 (25.0)	2 (25.0)	2 (25.0)	2 (25.0)	
Histological Grade, n(%)						
Well-differentiated	6 (27.27)	4 (66.67)	1 (16.67)	1 (16.67)	0 (0.0)	0.090
Moderately-differentiated	4 (18.18)	0 (0.0)	1 (25.0)	2 (50.0)	1 (25.0)	
Poor differentiated	12 (54.55)	4 (33.33)	4 (33.33)	0 (0.0)	4 (33.33)	
Tumor size (cm),						
< 5 cm	13 (34.21)	4 (30.77)	2 (15.38)	5 (38.46)	2 (15.38)	0.577
≥ 5 cm	19 (50.0)	9 (47.37)	6 (31.58)	2 (10.53)	2 (10.53)	
Myometrial Invasion, n(%)						
Negative	2 (5.26)	0 (0.0)	0 (0.0)	1 (50.0)	1 (50.0)	0.221
Positive (< / ≥ 50%)	36 (94.74)	16 (44.44)	9 (25.0)	7 (19.44)	4 (11.11)	
Mitotic index, n (%)						
< 15 per HPF	19 (63.33)	5 (26.32)	5 (26.32)	7 (36.84)	2 (10.53)	0.047
> 15 per HPF	11 (36.67)	8 (72.73)	2 (18.18)	0 (0.0)	1 (9.09)	
LVI, n (%)						
Positive	23 (67.65)	9 (39.13)	9 (39.13)	3 (13.04)	2 (8.70)	0.042
Negative	11 (32.35)	4 (36.36)	0 (0.0)	5 (45.45)	2 (18.18)	
Lymph Node status, n (%)						
Positive	15 (40.54)	3 (20.0)	5 (33.33)	4 (26.67)	3 (20.0)	0.032
Negative	22 (59.46)	13 (59.09)	4 (18.18)	4 (18.18)	1 (4.55)	
Pelvic wash, n(%)						
Positive	4 (10.81)	2 (50.0)	2 (50.0)	0 (0.0)	0 (0.0)	0.437
Negative	33 (89.19)	14 (42.42)	7 (21.21)	8 (24.24)	4 (12.12)	

^*^One-way Anova

The median age of all patients was 49 (40–55.5), with u-LMS patients found to have a median age group of 43 (39.5–52), HGESS with 53.5 (48–58), LGESS with 42.5 (37.5–55.5) and other histological subtypes (UUS, AS) with 56.5 (45–70). When compared, the differences in the age between the histological subtypes were highly significant in our analysis, with a *p*-value of < 0.001. Only two patients had a positive family history of cancer (one had a positive family of breast cancer, and the second had a positive family history of Ewing sarcoma). Seventeen patients (42.50%) were premenopausal, and 23 (57.50%) were postmenopausal. Most patients (37 cases, 91.43%) had a parity of ≥ 1. The menopausal status and parity were statistically significant characteristics among different histological subtypes, with a *p*-value of 0.039 and 0.004, respectively. Only 14 (35%) patients underwent initial uterine biopsy, out of which 13 had a positive diagnosis of malignancy. One patient had a negative initial biopsy; however, histopathological examination after primary surgery proved positive for malignancy. According to the FIGO stage, 16 patients (40%) had stage I, 8 (20%) had stage II, 5 (12.5%) had stage III, and 3 (7.5%) had stage IV disease (*p*-value = 0.034). Almost half of the patients (19 cases, 50%) had a tumor size of ≥ 5 cm, and the majority of the patients (36 cases, 94.74%) had a positive myometrial invasion. No significant differences were found between the histological subgroups concerning tumor size and myometrial invasion. The data on histological grade was available for 22 patients, out of which more than half of the patients (54%) were poorly differentiated. More than 15 mitoses per 10 HPF were found in 36.67% (n = 11/30) patients (*p*-value = 0.047). Lymphovascular invasion (LVI) was detected in 23 cases (68%), with u-LMS and HGESS groups having the highest no of positive LVI (*p*-value = 0.042).

Thirty-seven patients underwent primary surgical resection. Treatment and outcome characteristics are listed in Table 2.

**Table 2:** Treatment and Outcome characteristics of uterine sarcoma patients stratified on the histological type (*n* = 40)

**Characteristics**	**All patients (*n* = 40)**	**u-LMS** **(*n* = 16)**	**HGESS** **(*n* = 10)**	**LGESS** **(*n* = 8)**	**Other histologic subtypes** **(*n* = 6)**	***p*-value**
Primary surgery type, n(%)						
TAH + BSO	12 (30.0)	6 (50.0)	3 (25.0)	2 (16.67)	1 (8.33)	
TAH + BSO + PLND	15 (37.50)	3 (20.0)	5 (33.33)	4 (26.67)	3 (20.0)	0.045
TAH + BS + ovarian conservation	7 (17.50)	6 (85.71)	1 (14.29)	0 (0.0)	0 (0.0)	
Myomectomy	3 (7.50)	1 (33.33)	0 (0.0)	2 (66.67)	0 (0.0)	
Surgery not done	3 (7.50)	0 (0.0)	1 (33.33)	0 (0.0)	2 (66.67)	
Residual disease after primary surgery, n(%)						
Present	8 (21.62)	6 (75.0)	2 (25.0)	0 (0.0)	0 (0.0)	0.128
Absent	29 (78.38)	10 (34.48)	7 (24.14)	8 (27.59)	4 (13.79)	
Adjuvant chemotherapy received, n(%)						
Yes	24 (60.0)	7 (29.17)	6 (25.0)	5 (20.83)	6 (25.0)	0.123
No	16 (40.0)	9 (56.25)	4 (25.0)	3 (18.75)	0 (0.0)	
Chemotherapeutic regimen, n(%)						
Ifosfamide	1 (4.17)	1 (100.0)	0 (0.0)	0 (0.0)	0 (0.0)	
Doxorubicin	2 (8.33)	1 (50.0)	0 (0.0)	0 (0.0)	1 (50.0)	0.760
Doxorubicin + Ifosfamide	5 (20.83)	2 (40.00)	2 (40.00)	1 (20.0)	0 (0.0)	
Gemcitabine + Docetaxel	16 (66.67)	5 (31.25)	6 (37.5)	1 (6.25)	4 (25.0)	
Number of Cycles received n (%)						
3	5 (20.83)	1 (20.0)	2 (40.0)	0 (0.0)	2 (40.0)	
4	13 (54.17)	7 (53.85)	3 (23.08)	2 (15.38)	1 (7.69)	
6	6 (25.0)	1 (16.67)	3 (50.0)	0 (0.0)	2 (33.33)	0.311
Adjuvant Radiation Therapy, n(%)						
Yes	9 (22.50)	4 (44.44)	2 (22.22)	2 (22.22)	1 (11.11)	0.971
No	31 (77.50)	12 (38.71)	8 (25.81)	6 (19.35)	5 (16.13)	
Hormonal therapy, n(%)						
Yes	5 (12.50)	0 (0.0)	2 (40.0)	3 (60.0)	0 (0.0)	0.042
No	35 (87.50)	16 (45.71)	8 (22.86)	5 (14.29)	6 (17.14)	
Recurrences, n(%)						
Yes	18 (45.0)	5 (27.78)	4 (22.22)	5 (27.78)	4 (22.22)	0.327
No	22(55.0)	11 (50.0)	6 (27.27)	3 (13.64)	2 (9.09)	
Follow-up, n(%)						
Alive with disease	12(30.0)	4 (33.33)	2(16.67)	5 (41.67)	1 (8.33)	
Alive with remission	22 (55.0)	11(50.0)	6 (27.27)	3 (13.64)	2 (9.09)	0.049
Died	6 (15.0)	1(16.67)	2(33.33)	0 (0.0)	3 (50.0)	

Twenty-seven patients (67.50%) underwent total abdominal hysterectomy (TAH) and bilateral salpingo-oophorectomy (BSO), and among these, 15 patients (37.50%) also underwent pelvic lymph node dissection (PLND). Seven patients (17.50%) underwent TAH and bilateral salpingectomy (BS) with ovarian conservation. Three patients (7.50%) underwent myomectomy only. A significant association was found between these treatment groups and tumor histological subtypes (*p*-value of 0.045). Residual disease was found in 8 patients (21%) after primary surgery. A total of 24 patients (60%) received adjuvant systemic chemotherapy, with the majority of the patients (16 cases, 66.67%) having received a combination of gemcitabine and docetaxel. Five patients (21.83%) received a combination of doxorubicin and ifosfamide. However, three patients (12.5%) received single-agent systemic chemotherapy; 2 received doxorubicin, and one received ifosfamide. More than half (54%) of the patients received four cycles of systemic chemotherapy. Nine patients (22.50%) also received radiation therapy. Only five endometrial stromal sarcoma patients (22%) received hormonal therapy (*p*-value = 0.042). Follow-up revealed recurrence of the disease in 18 patients (45%). Among the available data, n = 9/18 patients (50%) had confirmed pelvic recurrences only. Out of these, seven patients underwent secondary surgical resection. Three patients had confirmed distant disease recurrence to the lung. The median follow-up duration was 25.7 months. At the last follow-up, 34 patients (85%) were alive (12 cases, 30%; with disease and 22 cases, 55%; without disease), and six patients (15%) had died (*p*-value = 0.049).

**Fig. 2:**
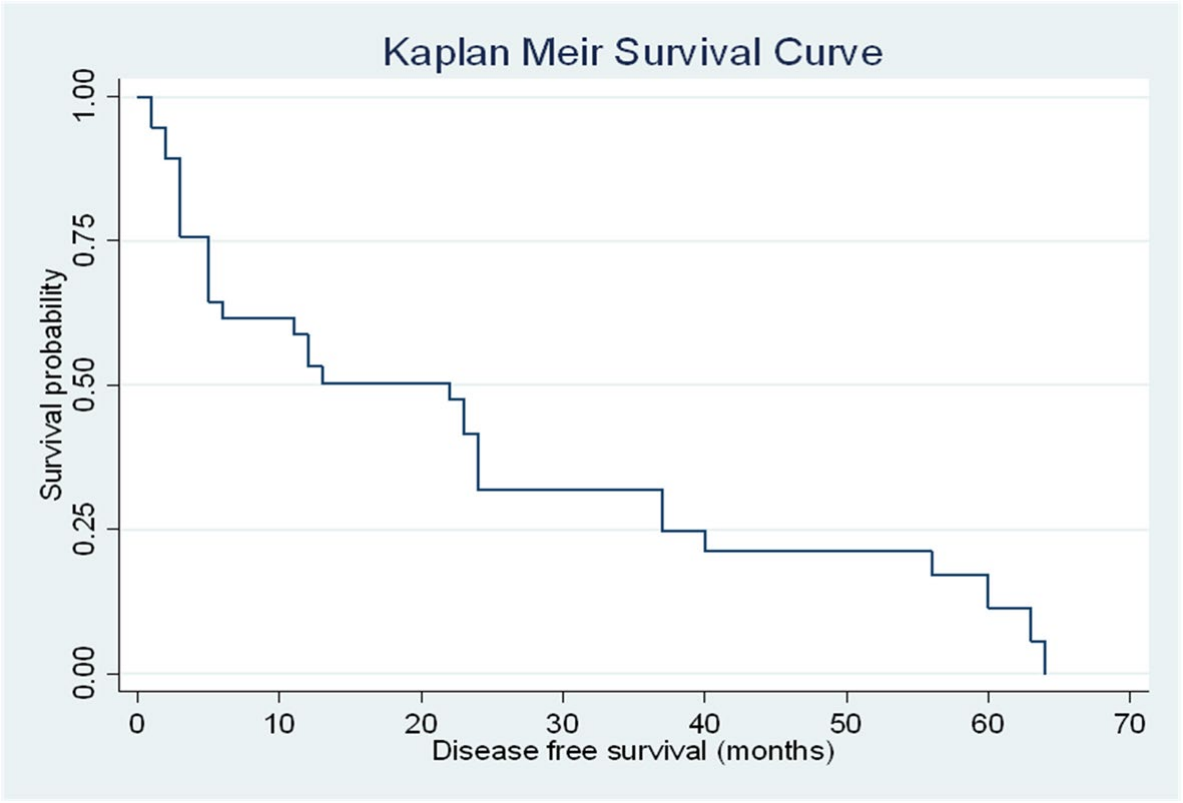
DFS for all patients

**Fig. 3:**
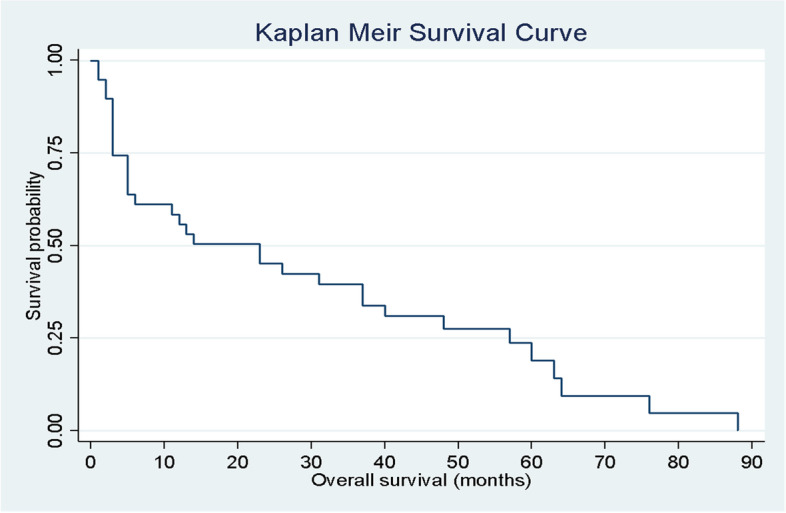
OS for all patients

The survival plots estimated by Kaplan–Meier analysis showed the DFS of 64 months and the OS of 88 months (*p*-value = 0.001) in the overall population (Figs. 2 and 3).

**Fig. 4:**
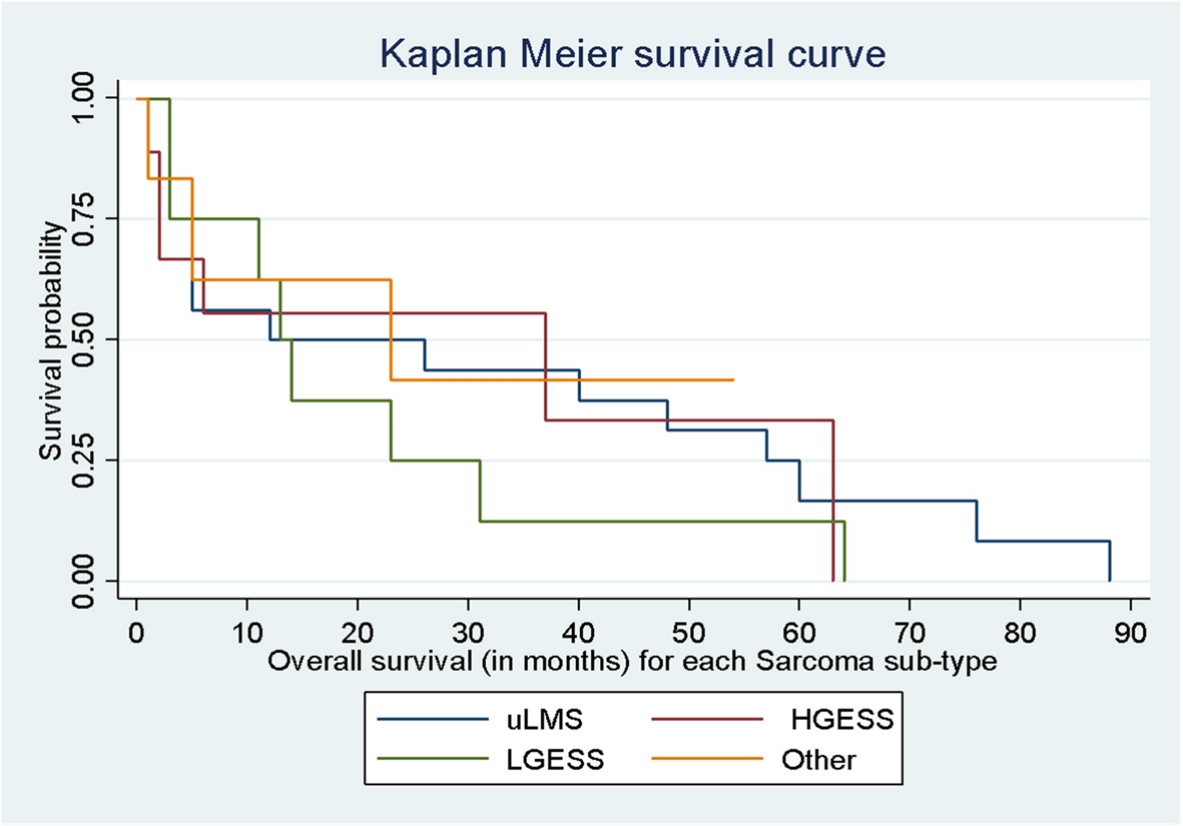
Median OS in each sarcoma subtype

The median DFS in all patients was 12 (range: 3–30) months, and the median OS was 14 (range: 3–76) months (*p*-value = 0.001) (Table 3). The median OS in each sarcoma subtype was 37 (range: 4–57.5) months in u-LMS, 19 (range: 2–46) months in HGESS, 13.5 (range:7–27) months in LGESS, and 14 (range: 3–23) months in others (UUS, AS) (*p*-value = 0.001) (Fig. 4) (Table 3).

**Table 3:** Disease-free survival and overall survival (in months) of uterine sarcoma patients stratified on the histological type (*n* = 40)

Characteristics	All patients (n)	u-LMS	HGESS	LGESS	Other histologic subtypes	*p*-value*
Disease-free survival (Median, IQR)	12 (3–30)	12 (4–32)	12 (2–37)	8 (3–23)	9.5 (3–22)	< 0.001
Overall survival (Median, IQR)	14 (3–76)	37 (4–57.5)	19 (2–46)	13.5 (7–27)	14 (3–23)	< 0.001

^*^Log-rank test

**Fig. 5:**
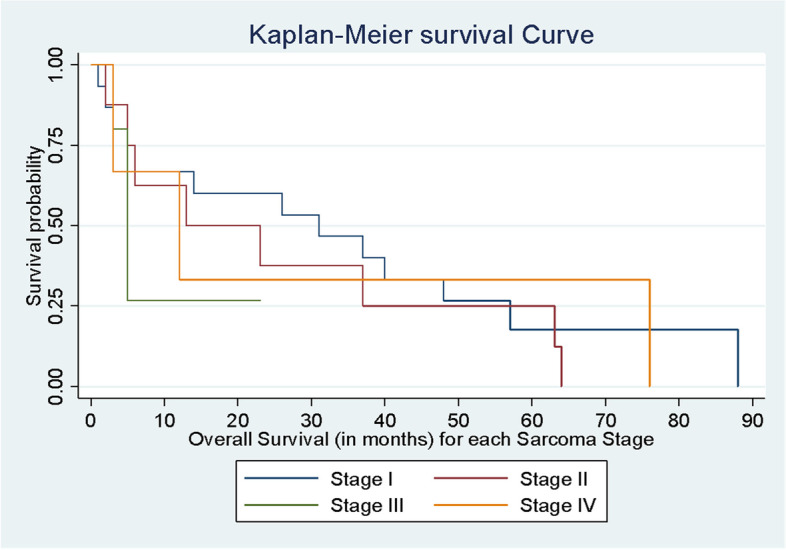
Median OS for each sarcoma stage

When the median OS was calculated based on the FIGO stage, it was found to be 31 (range: 3–54) months for stage I, 18.5 (range: 5–50) months for stage II, 13 (range: 3–30) months for stage III and 17 (4–46.5) months for stage IV (*p*-value = 0.001) (Fig. 5).

**Fig. 6:**
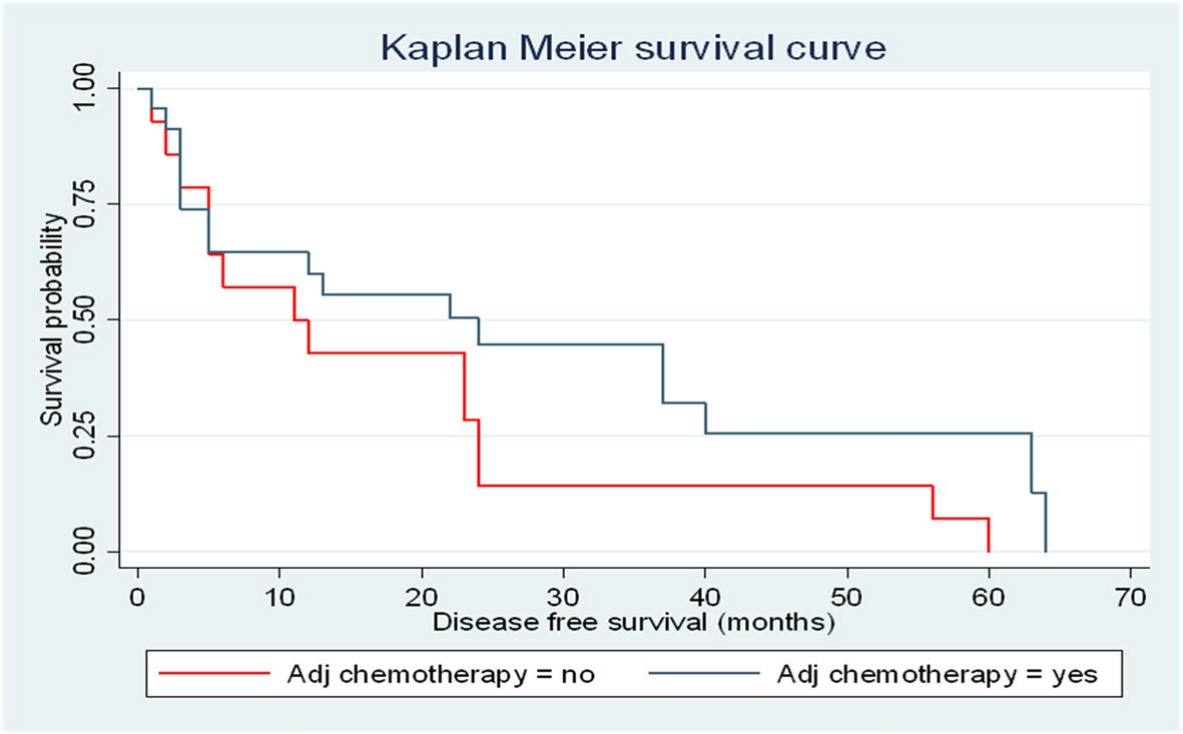
DFS in all patients with or without adjuvant systemic chemotherapy

**Fig. 7:**
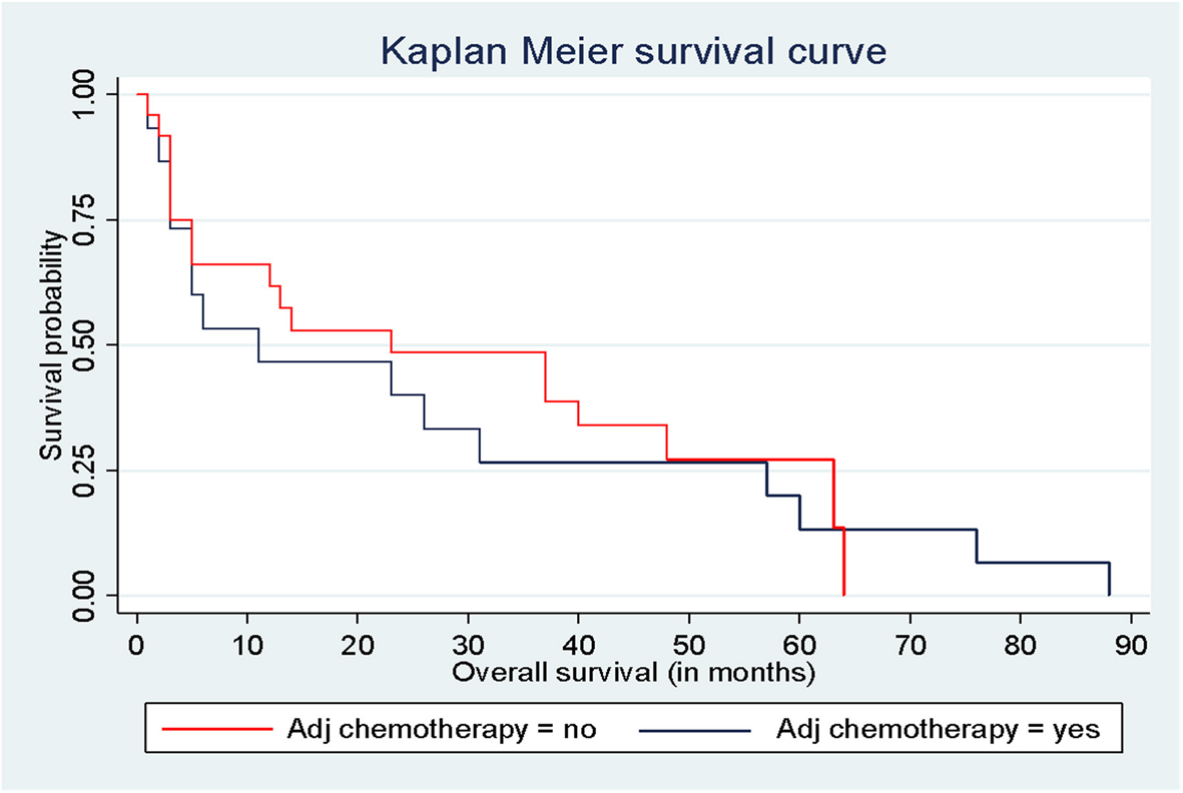
OS in all patients with or without adjuvant systemic chemotherapy

Our study also revealed a small, but significant DFS benefit in patients who received adjuvant systemic chemotherapy (with or without RT); median DFS of 13.5 (3–37) months compared to a median DFS of 11 (3–24) months in patients who did not receive adjuvant chemotherapy (*p*-value = 0.001). The median OS in patients who received adjuvant systemic chemotherapy (with or without RT) was 18.5 (range: 3–58) months, and it was 11 (range: 3–46) months in patients who did not receive adjuvant chemotherapy (*p*-value = 0.001). There was no difference in the OS in patients who received and did not receive adjuvant chemotherapy (with or without RT) (Figs. 6 and 7).

Cox regression analysis revealed that age, menopausal status, and parity had no significant effect on survival. However, larger tumor size and advanced FIGO stage were substantial factors in multivariate analysis associated with decreased survival in uterine sarcoma patients (Table 4).

**Table 4:** Multivariable analysis of uterine sarcoma patients reporting crude (cHR) and adjusted hazard ratios (aHR) with a 95% Confidence Interval (CI)

**Characteristic**	**cHR (95% CI)**	***p*-value**	aHR (95% CI)	***p*-value**
Age (years)				
< 50	1			
≥ 50	1.41 (1.16–2.78)	0.176*	1.36 (0.56–3.40)	0.091
Menstrual status				
Pre-menstrual	1			
Post-menstrual	1.23 (1.09–2.63)	0.081*	1.11 (0.67–2.99)	0.079
Parity				
Parity = 0	1			
Parity ≥ 1	1.76 (0.57–4.07)	0.300	-	-
Tumor size				
< 5 cm	1			
≥ 5 cm	1.95 (1.07–4.20)	0.020*	1.91 (1.39–2.82)	0.047**
Figo stage				
Stage I	1			
Stage II	3.43 (2.46–5.51)		2.51 (1.20–3.51)	
Stage III	4.55 (2.90–7.52)	0.002*	2.94 (1.43–4.07)	0.015**
Stage IV	4.24 (2.87–7.38)		2.81 (1.37–3.90)	
Grade of tumor				
Well	1			
Moderate	1.80 (1.12–3.56)	0.044	-	-
Poor	2.50 (1.91–5.33)			
LVI				
Negative	1			
Positive	0.61 (0.26–1.44)	0.273	-	-
Mitotic index				
< 15 per HPF	1		1	
> 15 per HPF	1.58 (1.16–3.63)	0.084*	1.49 (0.97–2.39)	0.052
Lymph node status				
Negative	1			
Positive	2.81 (0.89–6.81)	0.389	-	-

*Significant level on univariate analysis

**Significant level on multivariate analysis

## Discussion

Uterine sarcomas are extremely rare and belong to a heterogeneous group of malignancies. Most patients present with abnormal uterine bleeding, lower abdominal pain, and pelvic pressure [1, 2, 13, 30]. Understanding the development of uterine sarcomas has been gradual due to multiple histological subtypes, low incidence, and scarcity of data. Recent advancement has shown that specific chromosomal translocations resulting in gene fusion and transcription factor activation cause an increase in uterine sarcoma incidence and their different subtypes [2].

Personal history of malignancy, positive family history of cancer, and genetic factors are thought to play a role in the development of uterine sarcoma [16]. Prior exposure to pelvic radiation has been proposed as one of the possible causes of their development [2, 20]. However, in our study, only two patients had a positive family history of malignancy (breast cancer and Ewing sarcoma), and no patient had any personal history of sarcoma or other malignancy. Moreover, no patient in our study has a history of prior exposure to pelvic radiation. Nonetheless, the genomic landscape in the last few decades has revealed several genetic alterations in uterine sarcoma patients. These include genetic mutations in *TP53, RB1, ATRX, PTEN*, and *MED12* in u-LMS, fusion proteins *JAZF1–SUZ12* produced via chromosomal translocation; *t (7;17) (p15; q21)* in LGSEE and *YWHAE–FAM22* gene fusion in HGESS [21, 22].

Histopathological examination of our cohort of 40 patients with uterine sarcoma revealed u-LMS as the most frequent subtype, comprising 40% of the total included study subjects. This is also consistent with the published literature in which u-LMS was the most frequent subtype of uterine sarcoma [2, 23]. The median age in our study group was 49 (40–55.5 years), which is also similar to the previously published studies [23–25]. Previous studies on uterine sarcomas have included carcinosarcoma patients; in some, an almost equal number of carcinosarcoma and u-LMS patients were included in their analysis [23–26]. In contrast, our study did not include carcinosarcoma patients due to their epithelial origin and because they are no longer considered in the uterine sarcoma group [3]. This has provided a more relevant analysis of disease characteristics and outcomes knowing of the changes in the classification [23–25].

The utility of preoperative sampling is multifactorial. It not only helps in accurately diagnosing uterine sarcoma but also allows surgeons for appropriate surgical planning. In addition, stating these mesenchymal tumors' aggressive nature, careful evaluation for distant metastasis via systemic scans can be undertaken after preoperative diagnosis [28]. Unfortunately, only 14 patients in our study underwent preoperative biopsy, but 93% had a positive malignancy result. Similar results have been reported in the literature, in which 86–89% of preoperative biopsies revealed invasive malignancy in uterine sarcoma patients [27, 28].

TAH and BSO have been considered the most effective primary treatment for uterine sarcoma [13]. In our study, 27/40 patients underwent this standard surgical procedure. Almost half of them (55%) also underwent lymph node dissection. The data for PLND, in addition to TAH and BSO, is still evolving. The literature varies and may range from 30 to 74% [25, 27]. However, no significant survival benefits of PLND have been reported [25]. There is always a concern for ovarian conservation in young females undergoing gynecological surgical procedures. In patients with a limited disease to the uterus, ovaries of childbearing age women can be preserved [5, 29]. In our study, seven patients were amenable to ovarian conservation, with most (85%) belonging to the u-LMS group. However, such cases should be discussed in multidisciplinary tumor board (MDT) meetings, and risks vs. benefits should be discussed with the patients. In addition, the patient's tumor histology, hormone receptor status, menopausal state, and desire for fertility should be considered when performing an oophorectomy [30].

There is a scarcity of data evaluating chemotherapy or radiation after surgery in uterine sarcoma patients. In a clinical trial of 156 diagnosed cases of early-stage uterine sarcoma conducted by the Gynecological Oncology Group (GOG), postoperative doxorubicin decreased the recurrence rate compared to the observation (41 vs. 53 percent). However, no impact on PFS or OS was observed [31]. In another prospective trial of 25 women with stage I-IV u-LMS patients, a combination of gemcitabine and docetaxel resulted in a median PFS of 13 months in the entire cohort [32]. No conclusive impact on OS has been observed due to this study's lack of a control arm. In a prospective multi-center phase II trial of early-stage uterine sarcoma, docetaxel and gemcitabine combination followed by doxorubicin did not result in a lower recurrence rate or improvement in survival [33]. Similar results of no survival benefit have been observed in a real-world analysis of uterine sarcoma and carcinosarcoma patients [23, 25, 34]. In our study, 60% of the patients received adjuvant systemic chemotherapy, with the majority of the patients receiving a combination of gemcitabine and docetaxel. The median DFS was found to be 13.5 months in patients who received systemic chemotherapy compared to a median DFS of 11 months who did not receive adjuvant chemotherapy (*p*-value of 0.001). Consistent with the clinical trials, no difference in the OS was observed between the two groups on KM survival analysis. Our study also revealed the highest median DFS was observed in the u-LMS and stage I patients when survival was estimated based on histological subtypes and FIGO staging, which is compatible with the literature [25]. The survival seems to be the lowest in LGESS sub-group. This might be because the study subjects of LGESS are very less, comparative to u-LMS. The clinical impact of adjuvant RT on uterine sarcoma patients is uncertain, and it may depend on different histological subtypes [35]. No survival advantage has been reported with adjuvant RT in patients with uterine sarcoma [7]. The policy for the use of chemotherapy at our institute follows the NCCN guidelines [3]. At our center, cases of uterine sarcoma are discussed in the multidisciplinary tumor board (MDT) meetings and recommendations are made. For the majority of the patients with u-LMS, undifferentiated uterine sarcoma, HGESS and other rare uterine sarcoma patients with FIGO stage III-IV, we recommend four to six cycles of adjuvant systemic chemotherapy. The most common regimen used at our institute is combination systemic chemotherapy consisting of gemcitabine and docetaxel. However, anti-estrogen hormonal therapy is usually prescribed to LGESS patients with FIGO stage II-IV.

Furthermore, combined modality treatment with pelvic RT and chemotherapy remains an investigational approach [36]. Only nine patients in our study received RT; hence the number is too small to explain its clinical impact. Moreover, the data to support adjuvant hormonal treatment in uterine sarcoma is derived from retrospective studies, and it is mainly limited to endometrial stromal sarcoma. Adjuvant hormonal therapies have decreased the recurrence rate with no survival benefit in clinical trials [37–39]. Although a statistically significant difference (*p*-value = 0.042) was observed in patients who received adjuvant hormonal treatment in our study population, no conclusive results can be achieved because of the small number of these patients. With the advancement in precision oncology, biomarker-directed therapies targeting the tumor at the molecular level have been approved in advanced, recurrent uterine sarcoma patients. These include immunotherapies based on tumor mutational burden (TMB) and program death/program death ligand-1 (PD/PDL1) status and targeted therapies based on NTRK, BRCA, and homologous recombination gene status of the individual patient [40–42]. However, no such treatments have been approved for early-stage uterine sarcoma patients.

The overall prognosis of uterine sarcoma is poor, with a variable recurrence rate, and may range from 36 to 63% [10, 23–25, 27, 43]. Similarly, in our study, 45% of the patients had a disease recurrence with a median OS of 14 (3–76) months. There is no consensus on prognostic factors of uterine sarcoma. Some studies reported longer survival in younger patients [44]. However, several studies have not found any effect of age on survival outcomes [10, 45]. No statistically significant results of age on survival outcomes were observed in our study on multivariate analysis. Studies also reported a significant effect of the mitotic count, tumor grade, myometrial invasion, LVI, and tumor size on outcomes of uterine sarcoma [10, 24, 43, 46]. In our study, tumor size and FIGO staging significantly impacted survival on multivariate analysis. At the last follow-up, most of our study's patients (55%) remained alive and in remission. Few are alive with the disease (30%), while six patients (15%) have died.

Our study has certain limitations, as evidenced by the small number of patients. However, about the fact that uterine sarcomas are rare and it is conducted in a single institution, this study will add useful clinical information to the sparse literature on uterine sarcomas. Moreover, meticulous inclusion and exclusion criteria further limited the number of patients in our study. The results of our study should be interpreted in the equation to the limitations related to retrospective studies.

## Conclusion

Uterine sarcomas are rare malignancies with poor prognosis, even at early stages, with a high recurrence rate. Multiple factors, including tumor size, mitotic count, grade of the tumor, stage of the disease, LVI, and myometrial invasion, are associated with decreased survival and may vary among histological subtypes. Adjuvant chemotherapy, radiation therapy, and hormonal therapy may decrease the recurrence rate and improve disease-free survival but have no benefit on overall survival. However, the high rate of genetic alterations in uterine sarcoma and breakthrough advancements in targeted therapies may foster future probabilities of improved treatment modalities.

## Data Availability

The datasets are available from the corresponding author salman.soomar@aku.edu.

## Abbreviations

FIGO: International Federation of Gynecology and Obstetrics
uLMS: Uterine leiomyosarcoma
HGESS: High-grade endometrial stromal sarcoma
LGESS: Low grade endometrial stromal sarcoma
UUS: Undifferentiated uterine sarcoma
LVI: Lymphovascular space invasion
NCCN: National Comprehensive Cancer Network
TAH: Total abdominal hysterectomy
MMMT: Malignant mixed Mullerian tumors
